# Is CD1a involved in antitumour immune responses during carcinogenesis?

**DOI:** 10.1038/sj.bjc.6601597

**Published:** 2004-02-17

**Authors:** F Cappello

**Affiliations:** 1 Human Anatomy Section, Department of Experimental Medicine, University of Palermo, Italy

**Sir**,

I read with interest the article of Coventry and Morton (2003) that investigated DC infiltration within breast cancers and the association with survival. Interestingly, they found that more patients were alive at the 5-year time point in the group with higher CD1a DC density than the lower CD1a DC group, but this failed to reach statistical significance at the *P*=0.05 level.

In our opinion, the role of CD1 family molecules in antitumour immune responses, and in particular of CD1a, should be more debated, since its expression was recently described not only in monocyte-derived dendritic cells, but also in nonmesenchymal cytotypes, that is, epithelial cells (Ulanova *et al*, 2000).

We recently described the immunoexpression of CD1a by metaplastic cells in a large series of Barrett's metaplasia, both gastric- and intestinal-type (Cappello *et al*, 2003); we found a strong expression of this marker in epithelial cells of metaplastic glands, whereas normal gastric and colonic mucosae resulted negative. This expression had a diagnostic role, as well as predict a favourable outcome, since its expression lacked when, at follow-up, the same metaplastic lesions progressed towards dysplasia or cancer.

Based on all these observations, we strongly recommend further studies on the antitumour role of CD1a and its potential role during immuno-surveillance, not only in invasive cancers but also during the carcinogenetic steps.
